# Physician nutrition and cognition during work hours: effect of a nutrition based intervention

**DOI:** 10.1186/1472-6963-10-241

**Published:** 2010-08-17

**Authors:** Jane B Lemaire, Jean E Wallace, Kelly Dinsmore, Adriane M Lewin, William A Ghali, Delia Roberts

**Affiliations:** 1 Clinical Professor, Department of Medicine University of Calgary Health Sciences Center 3330 University Drive NW Calgary, Alberta, T2N 4N1, Canada; 2 Professor, Department of Sociology University of Calgary 2500 University Drive NW Calgary, Alberta, T2N 4N1, Canada; 3 Faculty of Kinesiology University of Calgary 2500 University Dr. NWCalgary, Alberta, T2N 4N1, Canada; 4 Department of Community Health Sciences University of Calgary TRW Building Ground Floor3280 Hospital Drive NW Calgary, Alberta, T2N 4Z6, Canada; 5 Professor of Medicine Department of Community Health Sciences University of Calgary TRW Building 3rd Floor3280 Hospital Drive NW Calgary, Alberta, T2N 4Z6, Canada; 6 Instructor Selkirk College 301 Frank Beinder Way Castlegar, British Columbia, V1N 4L3, Canada

## Abstract

**Background:**

Physicians are often unable to eat and drink properly during their work day. Nutrition has been linked to cognition. We aimed to examine the effect of a nutrition based intervention, that of scheduled nutrition breaks during the work day, upon physician cognition, glucose, and hypoglycemic symptoms.

**Methods:**

A volunteer sample of twenty staff physicians from a large urban teaching hospital were recruited from the doctors' lounge. During both the baseline and the intervention day, we measured subjects' cognitive function, capillary blood glucose, "hypoglycemic" nutrition-related symptoms, fluid and nutrient intake, level of physical activity, weight, and urinary output.

**Results:**

Cognition scores as measured by a composite score of speed and accuracy (Tput statistic) were superior on the intervention day on simple (220 vs. 209, p = 0.01) and complex (92 vs. 85, p < 0.001) reaction time tests. Group mean glucose was 0.3 mmol/L lower (p = 0.03) and less variable (coefficient of variation 12.2% vs. 18.0%) on the intervention day. Although not statistically significant, there was also a trend toward the reporting of fewer hypoglycemic type symptoms. There was higher nutrient intake on intervention versus baseline days as measured by mean caloric intake (1345 vs. 935 kilocalories, p = 0.008), and improved hydration as measured by mean change in body mass (+352 vs. -364 grams, p < 0.001).

**Conclusions:**

Our study provides evidence in support of adequate workplace nutrition as a contributor to improved physician cognition, adding to the body of research suggesting that physician wellness may ultimately benefit not only the physicians themselves but also their patients and the health care systems in which they work.

## Background

The typical work day of a hospital based physician is not only cognitively demanding, requiring complex decision-making in a fast-paced environment, it is also physically demanding, with extended hours and frequent on call periods. During their work time, physicians may be far removed from areas that provide access to nutrition (fluids and nutrients). As a result, physicians are often unable to eat and drink properly or at all during their work day [1-4].

Previous studies have demonstrated that the impairment of neurological functions such as fine motor skills, information processing, and memory, is linked to hypoglycemia and under-nutrition and may contribute to motor vehicle collisions and air crashes [5-9]. Conversely, sport scientists have demonstrated that optimized nutrition can sustain work output and concentration over extended periods of high physical and mental stress with great success. These techniques have been shown to improve health and wellness in occupational groups such as tree planters and heli-ski guides [10-13].

Physician performance has been increasingly linked to lifestyle and wellness factors such as sleep deprivation and stress and, in turn, to the quality of patient care [14-18]. However, there is a lack of research that empirically examines the impact of poor workday nutrition upon physicians' cognitive function, which is a foundation of their professional skill set. The objective of this study was to examine the effect of a nutrition based intervention, that of scheduled nutrition breaks during the work day, upon physician cognition, glucose, and hypoglycemic symptoms.

## Methods

### Setting and Participants

Twenty consecutive staff physician volunteers were recruited from the doctors' lounge of a large urban teaching hospital during the first week of May 2008 following a hospital wide poster campaign advertising study recruitment location and timing. The physicians selected two typical, similar work days to be scheduled as the baseline and intervention study periods during May and/or June 2008. All data were collected on site at the hospital. Ethics approval was obtained from the Conjoint Ethics Review Board of the University of Calgary. Written consent was obtained from participants.

### Study Design

This prospective study compared physicians' nutritional intake and cognitive function during work hours on two separate work days, a baseline day and an intervention day. A before and after study design was chosen rather than assigning participants to intervention or control days in random order, given the possibility that physicians assigned to first receive the intervention may be influenced to alter their typical nutritional habits. On the baseline day, the physicians followed their usual eating and drinking habits. On the intervention day, they were fed nutritious meals, snacks and fluids at scheduled intervals. Participants chose two typical and similar work days (in terms of workload, hours, sleep patterns, and other professional and personal commitments) within a two week period to serve as baseline and intervention days. Most physicians chose daytime work hours as the study period (17/20) while three chose evening and overnight work hours.

### Intervention

The intervention, that of ensuring that physicians consumed nutrients and fluids at regular intervals throughout their work day, was designed based on previous research where physicians and other health care professionals described barriers to achieving adequate nutritional intake during work hours [[1-4], unpublished data, Lemaire, Wallace, Dinsmore, Roberts]. The intervention had four key elements: providing healthy nutrition choices; enforcing nutrition breaks; maximizing ease of accessibility; and offering cost free nutrition. Nutrition provided during the study period on the intervention day was based on the recommendations of Canada's Food Guide [19] and a projected 24 hour total intake of 30.8 kcal/kg body weight with 15 percent of energy from fat, 15% from protein, and 70% from carbohydrate. On average, food and beverages were provided in six small meals, with selections offered based upon participant preference, and ease of storage, delivery and consumption in the hospital setting. This varied according to the number of hours worked by each participant and their individual nutritional choices. At each scheduled nutrition break, the research team contacted participants through hospital paging. Ready-to-consume and cost free nutrition was either waiting for physicians at the centrally located doctors' lounge or was brought to their practice location.

### Outcomes and Measurements

The primary outcome was cognition. Secondary outcomes were blood glucose levels and "hypoglycemic" nutrition-related symptoms. Baseline demographic characteristics were recorded at study enrolment. Fluid and nutrient intake and physical activity were measured on both days. At the beginning of each day, participants were weighed and fitted with an activity and heart rate monitor. At that time and at approximately two hour intervals on both days, measures of cognitive function, capillary blood glucose, "hypoglycemic" nutrition-related symptoms, food and fluid intake, and volume of urine excreted over the previous two hours were captured. Participants were weighed again at the end of each day. On the baseline day, the physicians maintained their usual eating and drinking habits. On the intervention day, the physicians reported to the study center fasting, and all nutrition for the day was delivered to the physician and recorded. The participants were blinded to their glucose and cognitive function test results at the time of testing.

#### Cognition

Cognition was measured using Brain Checkers software, Version 3.01 (Behavioural Neuroscience Systems LLC, Springfield MO) run on Palm Tungsten E2, (Palm Inc. Milpitas, CA). Two software programs were used. The simple reaction test was designed to measure the speed of motor response to a visual cue with repeated testing over thirty seconds. The complex reaction test, a choice reaction time and continuous performance task, was designed to measure running memory, attention and visual information processing with repeated testing over two minutes. This task requires the subject to indicate whether the current number (1 through 9 appearing randomly on a screen) matches the previously displayed number (with random time delay between the two) by tapping on the appropriate text box (labelled "same" or "different") located below the number. For both tests, the reaction times of each unique response as well as the mean reaction time for the session were recorded for each participant. Accuracy was documented in terms of percent correct responses, lapses (the subject did not respond to the stimulus) and impulses (the subject anticipated and acted before the prompt). A Tput statistic was calculated that captures the correct responses per minute of time available to respond. It represents a combination of speed and accuracy with a higher Tput statistic indicating a superior performance. Based on the manufacturer's recommendation, subjects completed three practice tests prior to baseline data collection. This approach eliminates any learning effect during the study period and prevents learning effects from confounding actual study measurements [20-22].

#### Glucose and "hypoglycemic" nutrition-related symptoms

Capillary blood glucose samples were collected from participants' fingertip and analyzed immediately using the Precision Xtra Blood Glucose Monitoring System (glucose measured in millimoles per liter). Participants were asked to report from a checklist of "hypoglycemic" nutrition-related symptoms, including those produced by falling glucose and counterregulatory hormones and by reduced brain glucose. The seventeen symptoms covered manifestations of adrenergic responses (sweating, sensation of warmth, anxiety, tremor or tremulousness, palpitations and tachycardia), glucagon responses (hunger, nausea), and neuroglycopenic responses (fatigue, dizziness, headache, visual disturbance, drowsiness, difficulty speaking, inability to concentrate, abnormal behavior, loss of memory, and confusion) [23,24]. The checklist response data were collapsed to a binary yes/no variable for the presence or absence of each symptom.

#### Hydration and nutrients

Body mass was measured using SR Model SR241 scales (accuracy = 0.2% ± 1 digit, resolution = 0.1 kg, SR Instruments, New Jersey). The measure of weight, performed by either of the two research assistants at the beginning and end of each study period, was standardized by using a single digital scale at the same location and ensuring participants' equivalent post urinary void state and clothing status (e.g. shoes off, pockets empty, pagers removed). Volume of fluid consumed and urine voided were quantified. Dietary analyses were performed using individual physicians' recorded diet history (instructions on how to record all food and drink consumption accurately were provided). Two-hour diet recall was also taken at each blood glucose sampling in order to enhance the validity of the dietary record. Only nutritional intake during the study period was analyzed using Diet Analysis+, Canadian version 4.0 (Wadsworth/Thompson Learning, Scarborough Ontario). Nutritional requirements were based upon the Dietary Reference Intakes (DRI 2002) [25], which reflect the current state of scientific knowledge.

#### Activity, patient load, stress and wellbeing

A triaxial accelerometer that records acceleration in three planes (Actiheart system, Mini Mitter Co. Inc, Bend OR) recorded activity level and heart rate simultaneously every fifteen seconds. Physicians were asked to rate both days on scales of 0 (low) to 10 (high) for workload, stress and general well being.

### Statistical Analysis

After determining the appropriateness of parametric analytical methods, the statistical significance of mean differences in blood glucose levels and cognitive test scores were calculated using a generalized estimating equation to take into account the repeated measurements taken during each study day; change in body mass, and fluid and nutrient intake on baseline and intervention days were assessed for normalcy and means were compared using paired two sided t-tests; where the assumption of normalcy was not met, results were presented as medians plus interquartile range, and compared using a Wilcoxon signed rank test. Variability in glucose values was calculated using the coefficient of variation (CV), which describes variability relative to the mean [CV = (standard deviation/mean)*100%]. Analysis of variance (ANOVA) was used to assess within-day differences in mean cognitive test scores across the sampling times. A Fisher's exact test was used to compare proportion of physicians reporting "hypoglycemic" nutrition-related symptoms on baseline and intervention days.

This study was originally conceived as a pilot study for preliminary testing of a nutrition based intervention, and for determination of multiple physiological and nutritional measurements in twenty working physicians. Given this, there were no a priori sample size considerations. However, based on mean glucose and cognition (Tput) values obtained, and corresponding standard deviations, we determined post-hoc that we had 96% power to detect a difference of 0.28 mmol/L in glucose values, and 97% power to detect a difference of 5 in Tput scores from the complex cognition test for the intervention day versus the baseline day.

All statistical analyses were performed using Stata 10 (StataCorp LP, College Station, Texas USA).

## Results

### Participant Characteristics

Twenty physicians from various medical specialties participated, with 10/20 participants (50%) from a medical specialty (General Internal Medicine, Hematology, Palliative Care, Intensive Care, Neurology, Diagnostic Imaging), 8/20 (40%) from a surgical specialty (Plastic Surgery, General Surgery, Ear Nose and Throat Surgery), and the remaining two (10%) from a primary care specialty (Family Practice, Hospitalist). The mean duration of medical practice was 16.5 years, ranging from 5 to 36 years. The mean age was 46.8 years, and ranged from 36 to 64 years, and 85% were male. The mean Body Mass Index was 25.5 kg/m^2 ^and ranged from 20.3 to 38.3 kg/m^2^. All participants were non-smokers and 15/20 (75%) reported exercising at moderate or high intensity for 30 minutes or longer at least 2-4 days per week. All twenty subjects completed both days with full data and follow-up for all study measures. No adverse effects of the intervention were reported.

### Glucose

**Figure 1:**
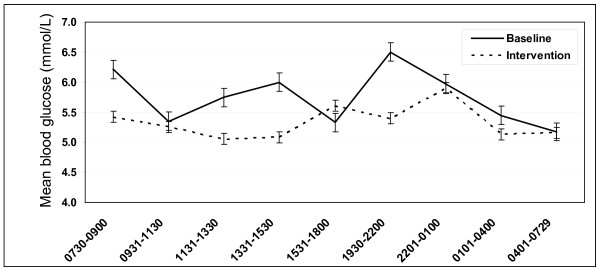
**Mean group glucose levels (with standard error) on baseline and intervention days**.

Group mean glucose was slightly lower and considerably less variable on the intervention day compared to the baseline day (Figure 1). The group mean glucose on the baseline day was 5.7 mmol/L, and dropped to 5.3 mmol/L on the intervention day (p = 0.03) (Table 1). The range decreased from 6.3 mmol/L at baseline to 4.1 mmol/L on the intervention day, indicating considerably less variability in glucose levels on the second day. This is also evident in the mean group coefficient of variation (CV), which decreased from 18.0% at baseline to 12.2% on the intervention day. The CV on the intervention day was lower than on the baseline day for 14/20 (70%) of participants, with individual decreases ranging from 0.1-28.2%. Analysis excluding the first glucose level of the day to account for fasting on the morning of the intervention day had little impact on overall results; thus results presented include all samples. Four physicians had serum glucose values in the hypoglycemic range of 3.3 to 3.8 mmol/L [23,24,26] on the baseline day, compared with two on the intervention day. The lowest value recorded in this study was 3.4 mmol/L, occurring in two subjects on the baseline day.

**Table 1:** Blood glucose levels, glucose variability, and reported "hypoglycemic" nutrition-related symptoms on baseline and intervention days.

	Baseline	Intervention	Difference	p-value
**Blood glucose (mmol/L)**				

Mean glucose (SE)	5.7 (0.1)	5.3 (0.1)	-0.3 (0.2)	0.03 ^a^

Minimum glucose value	3.4	3.6		

Maximum glucose value	9.7	7.7		

Range of glucose values	6.3	4.1		

				

**Variability in blood** **glucose values**				

Mean group coefficient of variation ^b^	18.0%	12.2%		

Smallest within-subject coefficient of variation	6.2%	4.5%		

Greatest within-subject coefficient of variation	33.7%	27.4%		

				

**Number of hypoglycemic symptoms** **reported by study participants**				0.36

0-1 discrete symptoms	40%	60%		

2-3 discrete symptoms	30%	15%		

4 or more discrete symptoms	30%	25%		

^a ^p-value for the difference in mean glucose (Intervention day-Baseline day)

^b ^Coefficient of variation = (SD/mean)*100%

### "Hypoglycemic" nutrition-related symptoms

Study participants often reported symptoms associated with "hypoglycemia" despite few glucose results in the hypoglycemic range. On the baseline day, the symptoms most commonly reported were hunger (15/20 participants), fatigue (9/20), sweating (6/20), sensation of warmth (6/20), and drowsiness (5/20). On the intervention day, the symptoms most commonly reported were hunger (12/20), fatigue (7/20), sweating (3/20), sensation of warmth (3/20), or inability to concentrate (3/20). Supplementary analysis of the number of individual symptoms reported by each participant was also undertaken. Although the results were not statistically significant (p = 0.36), there was a trend toward fewer symptoms during the intervention day relative to the baseline day (Table 1) with 60% versus 40% of participants reporting 0 or 1 discrete symptoms, 15% versus 30% reporting 2-3 discrete symptoms, and 25% versus 30% reporting 4 or more discrete symptoms. During the intervention day, 14/20 participants (70%) reported either fewer symptoms or no change compared to their reports at baseline.

### Cognition

Cognitive function at both simple and complex tasks as measured by the Tput statistic, and its components of reaction time, percent correct, lapses and impulses, indicated superior performance at every measure during the intervention day when compared to the baseline day (Table 2). For the simple reaction test, the group mean Tput statistic was superior (220 versus 209; p = 0.01) and the group mean reaction time was faster (279 msec versus 293 msec; p = 0.03). For the complex reaction test, the group mean Tput statistic was superior (92 versus 85; p < 0.001), the group mean reaction time was faster (606 msec versus 629 msec; p = 0.002), the percent correct was superior (98.2% versus 97.5%; p = 0.01), there were fewer mean lapses (4.3 versus 5.5; p = 0.05) and fewer mean impulses (4.5 versus 6.1; p < 0.001). When results were analyzed at the individual level, the majority of physicians demonstrated a superior Tput statistic on intervention day (13/20 or 65% for the simple reaction test, and 16/20 or 80% for the complex reaction test). To explore the possibility of a "learning effect" on cognitive performance, we tested for the presence of improved within-day performance on the test measures using ANOVA. The results revealed no significant difference in group mean scores across sampling times for simple or complex Tput statistics during the baseline (simple: p = 0.9; complex; p = 0.6) or intervention (simple: p = 0.8; complex; p = 0.9) days (results available upon request).

**Table 2:** Cognition measures: composite (Tput) and component scores on baseline and intervention days.

	Baseline	Intervention	Difference	p-value ^a^
**Simple reaction** **time test**				

Mean Tput ^b ^score (SE)	209 (6)	220 (6)	11 (4)	0.01

Mean reaction time in msec (SE)	293 (9)	279 (9)	-15 (7)	0.03

				

**Complex reaction** **time test**				

Mean Tput ^b ^score (SE)	85 (3)	92 (4)	7 (2)	< 0.001

Mean reaction time in msec (SE)	629 (18)	606 (21)	-23 (8)	0.002

Mean % correct (SE)	97.5 (0.4)	98.2 (0.3)	0.7 (0.3)	0.01

Mean lapses ^c ^(SE)	5.5 (0.9)	4.3 (1.0)	-1.2 (0.6)	0.05

Mean impulses ^d ^(SE)	6.1 (0.8)	4.5 (0.9)	-1.6 (0.4)	< 0.001

^a ^p-value for difference (Intervention day - Baseline day)

^b ^Tput is a composite score comprising reaction time and accuracy. Higher Tput scores indicate better performance.

^c ^Lapses: Subject did not respond to the stimulus.

^d ^Impulses: Subject anticipated and acted before the prompt.

### Hydration and nutrients

The physicians' nutrition, as measured by fluids and nutrients consumed, showed an improvement on the intervention day compared with their baseline nutrition practices. From the beginning to the end of the work day study period, (mean 9.7 ± 2.3 hrs baseline day and 10.4 ± 2.0 hrs intervention day as recorded by the activity monitors) participants showed a mean loss of body mass on the baseline day compared with a mean gain during the intervention day (-364 vs. +352 gms; p < 0.001). Mean fluid intake was significantly greater on the intervention day (1183 vs. 1358; p = 0.04) (Table 3). At the individual level, 14/20 (70%) of physicians had a decrease in body mass on the baseline day, whereas this occurred for only 6/20 (30%) on the intervention day. The majority of physicians (15/20 or 75%) consumed more fluids during the intervention than at baseline. Mean caloric intake was also significantly greater on intervention day compared with baseline day (935 vs. 1345 Kcal; p = 0.008) (Table 4). Analysis of macro- and micro-nutrients demonstrated increased absolute intake of protein, carbohydrate, polyunsaturated fats and fibre and decreased cholesterol on the intervention day. Low intake of fibre, potassium, calcium and vitamin D were also noted on the baseline day. Because the study periods did not encompass a full twenty four hours, it was not feasible to evaluate participants' nutrient consumption against the Dietary Reference Intake (DRI).

**Table 3:** Body mass and hydration parameters from the start to the end of the study period on baseline and intervention days.

	Baseline	Intervention	p-value
**Change in body** **mass in grams**			

Mean change (SD)	-364 (535)	+352 (499)	< 0.001

Greatest within-subject decrease	-1,360	-635	

Greatest within-subject increase	499	1043	

**Mean fluid intake** **in milliliters (SD)**	1183 (587)	1358 (595)	0.04


**Table 4:** Absolute macro- and micro-nutrient intake on baseline and intervention days, with dietary reference intakes.

		Baseline		Intervention		
**Nutrient**	**DRI ^a^**	**Mean** **intake^b^** **(SD)**	**Energy** **percent** **distribution^c^**	**Mean** **intake^b^** **(SD)**	**Energy** **percent** **distribution^c^**	**p-value** **for mean** **intake**

**Kcal**	2200-2500	935 (442)		1345 (414)		0.008

**Protein (g)**	46 - 56	35 (22)	15%	54 (18)	16%	0.01

**Carbohydrate (g)**	240-375	129 (66)	55%	192 (73)	57%	0.005

**Fat (g)**	49-90	35 (22)	34%	47 (16)	31%	0.08

**Saturated fat (g)**	< 24-25	13 (11)	13%	13 (7)	9%	0.94

**Polyunsat. fat(g)**	13-16	5 (3)	5%	9 (4)	6%	0.001

**Cholesterol (mg)**	300	137 (128)		123 (50)		0.66

**Fibre (g)**	21 - 31	8 (6)		23 (7)		< 0.001

**Total sugar (g)**	60-95	67 (42)		90 (29)		0.02

**Potassium (mg)**	4700	1261 (668)		2569 (775)		< 0.001

**Calcium (mg)**	1000-1200	575 (459) ^d^		679 (345) ^d^		0.07^d^

**Vitamin D (ug)**	15-25	0.5 (2.6) ^d^		1.1 (1.1) ^d^		0.71^d^

**Sodium (mg)**	1300	1266 (684)		1744 (940)		0.05

^a ^Dietary Reference Intake (values shown include the range for females and males)

^b ^Includes intake only during the study period and does not reflect 24 hour intake

^c ^Total exceeds 100% given difficulty of achieving perfect precision with recording of dietary intake.

^d ^Calcium and Vitamin D values are presented as median (IQR) and compared using the Wilcoxon Signed Rank test.

### Trial day similarity

Physicians were asked to choose two typical, similar work days for the study. Comparability was assessed using objective parameters of activity level and physicians' self reports of workload, stress, and general well being. There was no difference in the average number of hours worked during the study period comparing the baseline and intervention days (9.6 versus 10.4; p = 0.09). The group mean average heart rate (77 versus 74 beats per minute; p = 0.3) and physical workload (25 versus 22 activity counts per minute; p = 0.4) were also similar. Physicians' self reports on a scale of 0 (low) to 10 (high) comparing the baseline to the intervention day showed no difference for perceived workload (6.8 versus 6.9; p = 0.4), stress (5.0 versus 5.3; p = 0.3), or general well being (7.9 versus 7.7; p = 0.7).

## Discussion and Conclusions

The scheduled healthy food and fluids consumed during the intervention day were associated with improved physician cognition and less glucose variability. Although not statistically significant, there was also a trend toward the reporting of fewer hypoglycemic type symptoms. The change in cognitive function associated with the intervention appears notable relative to both population norms and age-related differences. For the simple reaction time test, the expected normal performance for ages 34-49 is a Tput score of 207, and a mean reaction time of 294 msec, and for ages 50-59, scores of 197 and 309 msec respectively. For the complex reaction time test, the expected normal performance for ages 34-49 is a Tput score of 100, and a mean reaction time of 547 msec and for ages 50-59, scores of 80 and 618 msec respectively [27]. Our study results show an association between the intervention and a numerical improvement in the test scores equivalent to the difference expected for norms across two age groups (i.e. 34-49 versus 50-59). This finding implies that adequate workplace nutrition may enhance cognitive function to the performance level of a younger age group. It is possible that the differences in cognition measures are simply due to random variation within normal limits, however the finding of superior cognitive performance on every parameter measured and the degree of change weigh against this.

The intervention was well received by participating physicians and its success may have been due to a number of factors. Nutrition was enforced through a scheduled regimen of food and fluid intake, readily available either at the centrally located doctors' lounge or at the physician's practice location. Physicians have previously indicated that they are often too busy to stop and eat and that limited access to nutrition during the work day, due to factors such as location, hours of operation, and cafeteria line ups, is a significant barrier [1]. They have also reported that their work ethic and professionalism (i.e. work and patients come first) are often factors that take priority over nutrition [unpublished data Lemaire, Wallace, Dinsmore, Roberts]. The intervention's key elements overcame these barriers. Additionally, the intervention provided healthy nutritional choices that were cost free. Given that limited food choices in terms of quality, appeal and variety are also perceived as obstacles to nutrition, it is not surprising that this also contributed to the intervention's success [[1], unpublished data Lemaire, Wallace, Dinsmore, Roberts]. Most physicians do not consider cost a major barrier [[1], unpublished data Lemaire, Wallace, Dinsmore, Roberts].

On a practical level, implementing a nutritional intervention in a health care system is feasible. Health care organizations are increasingly evaluating the quality of food and drink made available to patients and staff, recognizing the benefits of quality nutrition to overall health. Making time for nutrition can be encouraged by developing educational campaigns that promote the benefits of nutrition breaks, by carefully scheduling nutrition opportunities during work hours, and through peer support of nutrition as a necessary component of a physician's ability to deliver quality health care. While food and drink are already available at defined locations in most hospitals, improved access can be achieved through placement of nutrition stations in high workload areas. For example, healthy food stations may be set up near operating rooms and on acute care wards via mobile carts, or where physicians tend to gather most, such as in the doctors' lounge. Although the nutritional intervention in this study was cost free, qualitative interview data from the study participants (results available from authors) support that physicians are willing to pay for good quality food and drink, suggesting that most of these proposals would be cost neutral. Lastly, there may be a number of secondary benefits to health care systems that are staffed by physicians who are cognitively improved as a result of adequate nutrition.

As noted above, the literature provides evidence of the negative consequences of suboptimal nutrition for workers in a variety of work settings, in particular as they relate to cognitive function. Studies have also shown an association between the personal dietary habits of physicians and medical students and their nutrition counseling behaviors and attitudes [28,29]. Furthermore, a recent study by Tanaka et al found an association between poor dietary habits and the prevalence of fatigue in medical students [30]. The data from our study further document that physicians suffer from inadequate nutrition. We extend the body of research by describing physicians' nutrition on a typical work day, demonstrating an association between nutrition and cognitive function, and by exploring a potential intervention that can help to overcome the barriers to adequate nutritional intake for physicians in a hospital setting.

The main limitation of this study is the relatively small number of participants. However, our paired study design and richness of measures nonetheless permitted sufficient statistical power to detect several significant differences on key measures between the two study days. A second limitation is the predominantly male and hospital based physician study sample, with a potential lack of sampling across the different types of workdays physicians may experience. For further generalization of results, these variables, and others such as age and weight would also need to be taken into account in future research. A third potential limitation is the non-randomized study design. However, the study is not necessarily weakened by lack of randomization between the intervention and non-intervention control groups given we ensured comparability in our pair wise comparison within individuals. A fourth limitation is that the study was not designed to evaluate whether the differences found between the study days translate into improved patient care. Lastly, the manufacturers of the cognitive function tests cite research to support that the learner effect is attenuated after three practice trials. Although it is still possible that a learner effect influenced the study results, each participant did undergo three practice trials before starting the study, and the ANOVA analyses across sampling times within each study day did not suggest a learner effect for same days responses. Weighing against these limitations is the strength of the full participation of twenty physicians over both study days despite interruptions in their work day due to the extensive collection of physiological measures and the added time commitments.

Future studies might consider the development and randomized evaluation of an intervention that is more feasible in the acute care setting and that is based on a sustainable business model (e.g. mobile food carts that provide healthy nutrition at a reasonable cost). Our study provides evidence in support of adequate workplace nutrition as a contributor to improved physician cognition, adding to the body of research suggesting that physician wellness may ultimately benefit not only the physicians themselves but also their patients and the health care systems in which they work [31].

## Competing interests

The authors declare that they have no competing interests.

## Authors' contributions

All authors had access to the data, had a role in writing the manuscript, and approved the final manuscript. JL contributed to the study conception and design, acquisition of data, analysis and interpretation of data, drafting and critical revision of the manuscript, statistical analysis, obtaining funding, and administrative support. JW contributed to the study conception and design, analysis and interpretation of data, critical revision of the manuscript, statistical analysis, obtaining funding. KD contributed to the study design, acquisition of data, analysis and interpretation of data, critical revision of the manuscript and technical support. WG contributed to the analysis and interpretation of the data, critical revision of the manuscript, statistical analysis, and administrative and material support. AL contributed to statistical analysis and interpretation of data, drafting of the manuscript and critical revision of the manuscript. DR contributed to the conception and design of the study, acquisition of data, analysis and interpretation of data, critical revision of the manuscript, statistical analysis, and technical and material support. JL had full access to all the data in the study and takes responsibility for the integrity of the data and the accuracy of the data analysis, and had final responsibility for the decision to submit the manuscript for publication.

## Pre-publication history

The pre-publication history for this paper can be accessed here:

http://www.biomedcentral.com/1472-6963/10/241/prepub
